# Why do older adults hesitate to get the flu vaccine? A cross-sectional study on vaccine hesitancy in the post-COVID-19 era

**DOI:** 10.3389/fpubh.2025.1603091

**Published:** 2025-07-02

**Authors:** Xinyue Wang, Sanmei Wen, Jiaqian Wu, Ziyi Cui, Hong Shen, Shiyao Hu, Shujun Zeng, Yan Tang

**Affiliations:** ^1^Xuhui District Center for Disease Control and Prevention, Shanghai, China; ^2^School of Journalism and Communication, Tsinghua University, Beijing, China

**Keywords:** influenza, vaccination hesitancy, older adult, 3Cs model, vaccine literacy

## Abstract

**Objectives:**

To investigate the determinants of influenza vaccine hesitancy (VH) among older adults in Shanghai, China, using the 3Cs model (confidence, complacency, and convenience) and vaccine literacy (VL) framework. This study also explored the potential effect of COVID-19 vaccine hesitancy on influenza vaccine attitudes in the post-COVID-19 era.

**Methods:**

We conducted a community-based cross-sectional study from January to June 2024 in Shanghai, China, involving 1,300 adults aged ≥60 years. Participants were recruited through stratified random sampling. Inclusion criteria were: community-dwelling adults aged ≥60 years in Xuhui District. Multinomial logistic regression models were used to identify predictors of vaccine hesitancy, adjusting for sociodemographic variables, self-reported health status and self-report vaccination experiences. Structural equation modeling (SEM) was employed to examine the underlying factors contributing to vaccine hesitancy and quantify their interrelationships.

**Results:**

A high proportion (85.2%) of participants exhibited influenza vaccine hesitancy, with 16.2% being complete refusers. Key predictors of hesitancy included distrust in vaccine efficacy (adjusted Odds Ratio [aOR] = 2.28 for refusal), low perceived influenza severity (aOR = 5.59 for refusal), and overreliance on non-pharmaceutical interventions (NPIs) (aOR = 3.37 for refusal) and influenza-specific medication (aOR = 3.76). Limited health communication with community health workers (CHWs) and low family support significantly amplified refusal risks (aOR = 3.63). Higher vaccine knowledge reduced hesitancy (aOR = 1.85), though paradoxically, higher critical vaccine literacy correlated with refusal tendencies (aOR = 0.36). Significant standardized estimated coefficient (*β*) were observed between confidence and complacency (*β* = 0.846), side-effect experience and complacency (*β* = 0.293), side-effect experience and depression (*β* = 0.294), convenience and depression (*β* = 0.293), and side-effect experience and needle phobia (*β* = 0.362).

**Conclusion:**

Vaccine confidence deficits and complacency regarding influenza severity are major drivers of hesitancy in older adults. This hesitancy is further exacerbated by COVID-19 vaccine skepticism and nuanced aspects of vaccine literacy. System-level interventions should integrate proactive vaccine counseling into routine care, strengthen family engagement in immunization decisions, and develop misinformation-resilient vaccine literacy programs specifically tailored for this vulnerable population.

## 1 Introduction

Influenza is a significant global health concern, causing seasonal epidemics that result in substantial morbidity and mortality each year (1). According to the World Health Organization (WHO), seasonal influenza affects approximately 1 billion people annually, with 3–5 million cases of severe illness and 290,000–650,000 influenza-related respiratory deaths worldwide (60). Older adults are particularly vulnerable, as most influenza-related deaths occur in this population due to weakened immune responses and the exacerbation of chronic conditions such as cardiovascular disease, diabetes, and chronic obstructive pulmonary disease (COPD) (2–4). The risks associated with influenza infection have been further compounded in the post-COVID-19 era, as studies have shown that co-infection with COVID-19 and influenza significantly increases patient mortality and the likelihood of requiring invasive mechanical ventilation (5, 6). Given these concerns, influenza vaccination remains the most effective preventive measure, reducing the risk of severe disease, hospitalization, and death, particularly among high-risk populations.

Despite WHO recommendations that at least 75% of older adults receive annual influenza vaccination, global vaccination coverage remains suboptimal, with substantial regional disparities. In high-income countries such as the United States, influenza vaccination coverage among the older adult reached 80.9% in 2021 (58). While Japan reported a 67.32% vaccination rate among older adults in 2020 (7), South Korea has consistently maintained coverage above 80% due to longstanding government-supported free vaccination programs (8). However, China remains an outlier, with an overall influenza vaccine coverage rate of only 2.47% among older adults in 2021 (9, 10). In Shanghai, the vaccination rate among the older adult was even lower, at 0.4% (11), despite influenza’s well-documented burden in this population. During the COVID-19 pandemic, influenza vaccination coverage among the older adult in Shanghai rose to 4.1%, a rise that has been partially attributed to heightened public health campaigns promoting COVID-19 vaccination (12).

Vaccine hesitancy (VH), defined as the delay or refusal of a vaccine despite the availability of vaccination services (13), is a major contributor to low influenza vaccination rates and has been recognized by WHO as one of the top ten global health threats (59). Studies have identified multiple factors influencing VH, including socioeconomic status (14), education level (15), trust in the healthcare system (16). However, psychological factors, such as depression (17, 18), cognitive function (19), and perceived disease risk (20) are increasingly recognized as influencers of health decision-making, including vaccination behavior. Several theoretical frameworks have been proposed to explain VH, such as the health belief model (HBM), which emphasizes perceived risks and benefits, and the theory of planned behavior model (TPB), which considers social norms and behavioral intentions (21, 22). However, these models do not fully capture the complex interplay of factors influencing VH among older adults. To address this, the WHO’s Strategic Advisory Group on Experts (SAGE) proposed the 3Cs model, which provides a more comprehensive framework for analyzing VH. The model classifies VH into three dimensions: confidence (trust in vaccine safety, efficacy, and policymakers), complacency (low perceived risk of the disease, self-efficacy and the ability to act), and convenience (accessibility and affordability of vaccination services) (23). The 3Cs model delineates the scope of VH, and helps analyze the causes and influences of VH among people of different age groups in different contexts (23).

Moreover, in recent years, scholars have developed the concept of vaccine literacy (VL), a component of health literacy, which may be a promising approach to exterminating VH (24). VL extends beyond basic knowledge of vaccines to encompass an individual’s ability to access, comprehend, evaluate, and apply vaccine-related information in decision-making (25). Studies have shown that higher levels of VL are associated with increased vaccine acceptance, as observed in HPV vaccination among college students and COVID-19 vaccine uptake among adults (26, 27). However, despite its theoretical importance and the significant challenges, older individuals often face in accessing and interpreting health information, specific investigations into the levels and influence of influenza vaccine literacy among older adult Chinese populations are notably scarce. The Health Literacy about Vaccination of Adults in Italian (HLVa-IT) was constructed by Biasio et al., aiming to assess vaccination-related health literacy among adults in Italy (28). It includes three scales: functional VL (language capabilities, involving the semantic system), interactive VL (the capacity to proactively acquire, interpret, and engage in information) and critical VL (problem solving and decision making) (28). Currently, it is the most extensively employed tool within this field of research (27). Given the particularly low influenza vaccine uptake among older adults in China, further investigation into the relationship between VL and VH is warranted to inform targeted public health interventions.

Given the alarmingly low influenza vaccination coverage in China’s older adult population, a deeper understanding of its underlying causes is urgently needed. While previous studies have examined VH and VL in various populations, a comprehensive exploration of the specific factors influencing influenza VH among older adults within the unique post-COVID-19 context, particularly encompassing vaccine literacy, remains limited. This study aims to fill this critical gap by applying the 3Cs model to analyze influenza VH among older adults in China, beyond the scope of COVID-19. Additionally, it is the first to use the HLVa-IT scale, which was validated for the Chinese population in 2023 (29), to evaluate the relationship between VL and influenza VH among older adults. Findings from this research may provide critical insights for designing evidence-based public health strategies to optimize influenza vaccination policies and enhance vaccine coverage among China’s aging population.

## 2 Methods

### 2.1 Study design

**Figure 1:**
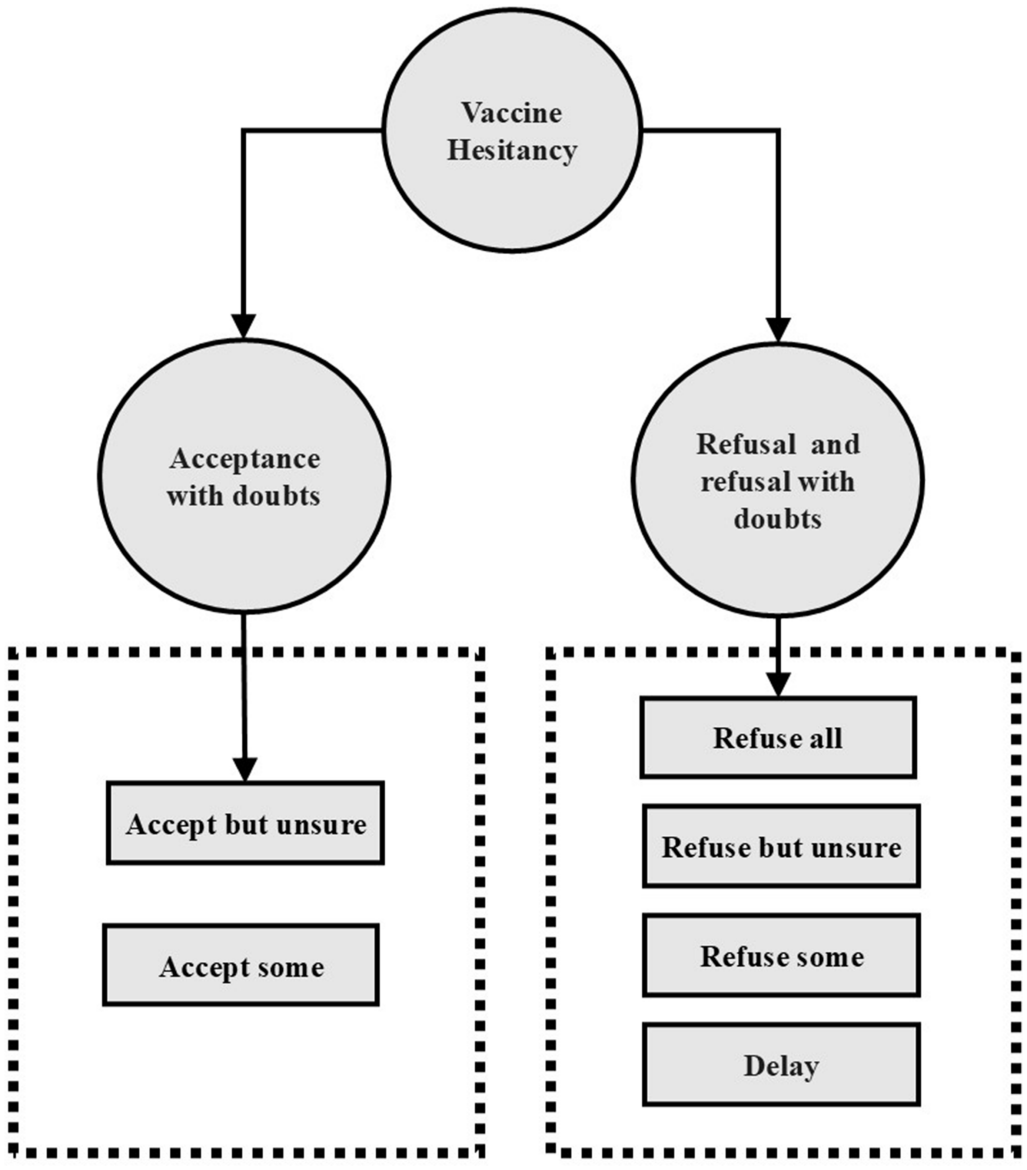
Theoretical model diagram based on WHO definition of vaccine hesitancy.

This study employed a multicenter cross-sectional design to assess influenza VH among older adults in Shanghai, China. Figure 1 displayed a theoretical model diagram based on WHO definition of vaccine hesitancy, referring to delay in acceptance or refusal of safe vaccines despite availability of vaccination services (30). Following the WHO definition of VH, participants were asked to indicate their willingness to receive the influenza vaccine by responding to the question: “Are you willing to receive the influenza vaccine?” The response options were: “Refuse all,” “Refuse but unsure,” “Refuse some,” “Delay,” “Accept some,” “Accept but unsure” and “Accept all,” a 7-point scale recommended by the SAGE working group on vaccine hesitancy (23). “Refuse all” is an extreme form of vaccine hesitancy, which is also classified as vaccine hesitant in many studies (31–33). In order to conduct more in-depth research, this study further subdivided vaccine hesitant people into vaccine acceptance with doubts and vaccine refusal and refusal with doubts. (see Figure 1) “Refuse all,” “Refuse but unsure,” “Refuse some” or “Delay” were categorized as vaccine refusal and refusal with doubts, indicating reluctance or refusal toward vaccination despite some level of concern. Similarly, participants who selected “Accept some” or “Accept but unsure” were considered as vaccine acceptance with doubts, meaning they were willing to receive the vaccine but still harbored uncertainties. Those who chose “Accept all,” corresponding to a direct acceptance, were categorized as non-hesitators, signifying complete vaccine acceptance without concerns. Using the same classification approach, we also assessed participants’ hesitancy toward COVID-19 vaccination.

A structured questionnaire assessed VH, 3Cs model components (confidence, complacency, convenience) (see Table 1), VL via the Health Literacy about Vaccination in Adults (HLVa-IT) scale (see Table 2). The Geriatric Depression Scale (GDS-15), a validated tool for assessing depression in older adults (34, 35) (Cronbach’s alpha ≈ 0.80), was used to measure depressive symptoms, with scores ≥5 indicating potential depressive symptoms, but not a definitive diagnosis. Previous research defines complacency as encompassing low perceived risk of contracting influenza, limited knowledge of influenza and its vaccine, and prejudices against vaccination (36). Our questionnaire retained core elements of complacency, including perceived severity (Items 1–3) and susceptibility (Items 4–5) to influenza and co-infection. To better capture beliefs prevalent among older adults, we added four items (Items 6–9): reliance on influenza-specific medications, belief in personal immunity, perceived lack of necessity for older adult vaccination, and trust in non-pharmaceutical interventions (NPIs) like masks based on previous research (37). Additionally, the survey incorporated other potential determinants of influenza VH, including engagement with community health workers (e.g., family doctor sign-up rates, frequency of vaccine-related health communication), history of chronic illnesses and falls, allergy history, exposure to negative vaccine-related information and previous experiences with vaccination. A supplementary questionnaire was developed to assess general vaccine knowledge among older adults, with a specific focus on common misconceptions about influenza vaccines, adapted from prevalent online misinformation sources. Additionally, VL was assessed using the HLVa-IT tool, which evaluates functional, interactive, and critical VL (see Table 2). Scores were calculated based on the mean values of responses, ranging from 1 to 4. Higher scores on functional VL indicate higher levels of vaccine literacy, whereas higher scores on interactive and critical VL are associated with lower levels of vaccine literacy.

**Table 1:** Questions used to measure 3Cs factors in the survey instrument.

Factors	Questions
Willingness	Item 1: Are you willing to receive the influenza vaccine?
Complacency	Item 1: What do you think of the severity of being infected with influenza?
	Item 2: What do you think of the severity of being infected with influenza and COVID-19 at the same time?
	Item 3: What do you think of the severity of spreading influenza to family?
	Item 4: What do you think of the probability of being infected with influenza?
	Item 5: What do you think of the probability of being infected with influenza and COVID-19 at the same time?
	Item 6: Since there are drugs for influenza, there is no need to get the vaccine.
	Item 7: There was no need to get the influenza vaccine because I had immunity.
	Item 8: Vaccination is not necessary for the older adult because of the high age.
	Item 9: Since NPIs can prevent infection, there is no need to get the vaccine.
Confidence	Item 1: What do you think of the skills and competencies of health workers who administer vaccines?
	Item 2: What do you think of the efficacy of influenza vaccines?
	Item 3: What do you think of the safety of influenza vaccines?
	Item 4: What do you think of the efficacy of COVID-19 vaccines?
	Item 5: What do you think of the safety of COVID-19 vaccines?
Convenience	Item 1: I can get the influenza vaccine independently if I wish.
	Item 2: My family encouraged me to get the influenza vaccine.
	Item 3: What do you think of the frequency of community health activities?
	Item 4: Community health workers regularly communicate with me about influenza vaccines.
	Item 5: I have an assigned family physician.
	Item 6: I’ve heard neighbors talk about adverse vaccine reactions.
	Item 7: The price of the vaccine made me reluctant to receive the flu vaccines.
	Item 8: I think the current opening hours of the influenza vaccine service are appropriate.

Responses were recorded using a five-point Likert scale, ranging from “Very low” to “Very high” for severity/probability perception questions and from “Strongly disagree” to “Strongly agree” for attitudinal statements.

**Table 2:** Questions used to measure health literacy about vaccination in the survey instrument.

Factors	Questions
Functional vaccine literacy	Item 1: Did you find the material as a whole text or image difficult to read?
	Item 2: Did you find words you did not know?
	Item 3: Did you find that the texts were difficult to understand?
	Item 4: Did you need much time to understand them?
	Item 5: Would you need someone to help you understand them?
Interactive vaccine literacy	Item 1: Have you consulted more than one source of information?
	Item 2: Did you find the information you were looking for?
	Item 3: Did you understand the information found?
	Item 4: Have you had the opportunity to use the information?
	Item 5: Did you discuss what you understood about vaccinations with your doctor or other people?
Critical vaccine literacy	Item 1: Did you consider whether the information collected was about your condition?
	Item 2: Have you considered the credibility of the sources?
	Item 3: Did you check whether the information was correct?
	Item 4: Did you find any useful information to decide on whether or not to get vaccinated?

Responses were recorded using a four-point Likert scale, ranging from “Never” to “Often”.

Lastly, a pilot survey was conducted with 30 older adults to evaluate the clarity and readability of the questionnaire, with minor modifications made based on feedback. The final survey required about 15 min for completion.

### 2.2 Sample size and data collection

According to the Law of the People’s Republic of China on the Protection of the Rights and Interests of the Elderly (38), citizens aged 60 and above are defined as older adult in China. A stratified random sampling approach was used to ensure a representative sample of older adults (≥60). The study was conducted in the Xuhui district, the largest urban district in Shanghai, China from January to June 2024. Participants were randomly recruited through a community-based management system, an official registry that includes residents of all ages and is commonly used for sampling in population-based surveys. We set the following inclusion Criteria: (1) Community-dwelling adults aged ≥60 years in Xuhui District; (2) Permanent residency status (≥6 months residence in Shanghai during the preceding 12 months, irrespective of household registration); (3) Preserved cognitive capacity with ability to provide informed consent; (4) Verbal competence for clear self-expression; (5) Voluntary participation. Exclusion Criteria: (1) Diagnosed cognitive impairment or severe psychiatric disorders; (2) Decompensated chronic conditions (cardiac failure, respiratory failure, etc.); (3) Functional dependency; (4) Restricted mobility. Prior to survey participation, all respondents provided written informed consent. This document explicitly assured that: (1) All responses would remain strictly confidential through anonymized data processing; (2) Participation would entail no negative consequences for individuals; and (3) The study involved no human experimentation. Collected data served exclusively to assess vaccine literacy levels among older adult residents in Shanghai and inform evidence-based health policy development.

A total of 1,313 older adult individuals aged ≥60 years were initially recruited. Before data collection, 26 trained enumerators from 13 community centers received standardized training to address inquiries during the administration of questionnaires for the older adult. Surveys were conducted via face-to-face interviews, during which enumerators provided clarification on survey questions and directly entered responses into an electronic questionnaire platform.^1^

The questionnaire was designed to take a minimum of 15 min to complete. Of the 1,313 recruited participants, four declined to participate, and nine participants failed to finish the questionnaires. A total of 1,300 fully completed questionnaires were included in the final analysis. The required sample size was estimated using the standard formula for cross-sectional studies with an *α* error of 0.05 and a permissible margin of error (*δ*) of 0.05:


$$N=\frac{{\mu_{\alpha }}^{2}\times p(1-p)}{\delta^{2}}\times \text{deff}$$

*N* represents the sample size, *μ* is the standard normal deviation of *α*, *μ_α_* = 1.96 when *α* error of 0.05, and p represents the estimated prevalence of influenza VH. Based on a prior study, the influenza VH rate in mainland China was 37.18%, which informed the sample size calculation (39). Design effect (deff) equals 2 in this study.

### 2.3 Statistical analysis

We performed factor analysis to verify the reliability and validity of each questionnaire scale. Confirmatory Factor Analysis (CFA) was employed to validate the predefined factor structure. Items demonstrated standardized factor loadings >0.50, exceeding the minimum threshold, were considered as acceptable item retention. Composite reliability (CR) > 0.7 and squared multiple correlations (SMC) > 0.4 were considered as high aggregation validity (40). Model fit indices can further confirm structural validity (e.g., CFI > 0.90, RMSEA < 0.08). Additionally, Cronbach’s alpha coefficients exceeding 0.70 were considered as robust internal consistency reliability (41).

Descriptive statistics were used to summarize participant characteristics and VH status. The chi-square test was applied to examine the associations between influenza VH and key determinants, including socio-demographics, the 3Cs model components, VL levels, and other potential influencing variables. Multinomial logistic regression was performed to identify independent predictors of VH, with “No hesitancy” serving as the reference group, adjusting for confounders including sociodemographic variables, self-report health status and self-report vaccination experiences. After that, we employed structural equation modeling (SEM) to examine the underlying factors contributing to vaccine hesitancy and quantify their interrelationships. SEM assumed the effect of 3C factors as three latent variables on influenza VH combined with other factors screened by multinomial logistic regression. After fineness optimization and further modifications, the final integrated model for influenza VH was established.

We also used Stepwise Regression and calculated the Variance Inflation Factor (VIF) for all predictor variables to identify and eliminate variables that did not contribute much to the model, thus reducing the effect of multicollinearity. Factors with VIF values greater than 10 imply multicollinearity and will be excluded from the model. Adjusted Odds ratios (aORs) and 95% confidence intervals (CIs) were reported for each included determinant. A two-sided *p*-value <0.05 was considered statistically significant. Data analyses were conducted using R software (version 4.4.2).

## 3 Results

### 3.1 Reliability and validity of items

Based on stepwise regression results and the values of VIFs, we excluded several variables due to multicollinearity and model fit concerns: COVID-19 vaccine hesitancy, Confidence items 4–5, and Convenience items 7–8. For the retained 3Cs model items, standardized factor loadings ranged from 0.642 to 0.887, greater than the 0.50 threshold (see Table 3). Model fit indices (CFI = 0.921, TLI = 0.901, RMSEA = 0.097, SRMR = 0.075) were marginally acceptable. Cronbach’s *α* of confidence, convenience and complacency were 0.889, 0.841, and 0.754, respectively, all exceeding the 0.70 reliability standard. For the HLVa-IT scale adapted for older adult populations, standardized factor loadings spanned 0.719–0.917 (all > 0.50) (see Table 3). Model fit indices (CFI = 0.947, TLI = 0.935, RMSEA = 0.097, SRMR = 0.042) were acceptable fit. Both scales demonstrated robust structural validity and internal consistency (Table 4).

**Table 3:** The reliability and validity of items.

Dimension	Factors	Factor loading Std.	SMC	CR	Cronbach’s а
The 3Cs factors	Complacency			0.89	0.889
	Item 1	0.635	0.403		
	Item 2	0.661	0.437		
	Item 3	0.692	0.479		
	Item 4	0.767	0.589		
	Item 5	0.697	0.486		
	Item 6	0.679	0.462		
	Item 7	0.697	0.485		
	Item 8	0.654	0.428		
	Item 9	0.706	0.499		
	Confidence			0.851	0.841
	Item 1	0.642	0.412		
	Item 2	0.887	0.787		
	Item 3	0.883	0.78		
	Convenience			0.753	0.754
	Item 1	0.600	0.410		
	Item 2	0.729	0.531		
	Item 3	0.796	0.634		
Vaccine literacy	Functional			0.93	0.93
	Item 1	0.823	0.677		
	Item 2	0.89	0.792		
	Item 3	0.894	0.8		
	Item 4	0.834	0.695		
	Item 5	0.822	0.676		
	Interactive			0.924	0.921
	Item 1	0.779	0.608		
	Item 2	0.899	0.808		
	Item 3	0.917	0.84		
	Item 4	0.887	0.787		
	Item 5	0.719	0.516		
	Critical			0.939	0.939
	Item 1	0.885	0.782		
	Item 2	0.894	0.798		
	Item 3	0.896	0.802		
	Item 4	0.89	0.793		

Std., standardized estimate; SMC, squared multiple correlations; CR, composite reliability.

**Table 4:** Fit indices of the integrated model for influenza vaccine hesitators.

Fit indices	Acceptable range	Measured value of vaccine hesitancy	
		Acceptance with doubts	Refusal and refusal with doubts
Degrees of freedom (df)	–	31.283	41.921
Chi-square (*χ*^2^)	–	12	16
*χ*^2^/df	<3.00	2.607	2.620
GFI	≥0.90	0.9987	0.993
CFI	>0.90	0.977	0.993
NFI	>0.90	0.965	0.988
RMR	<0.05	0.009	0.004
SRMR	<0.08	0.024	0.011

### 3.2 Characteristics of respondents

One thousand three hundred older adult participants completed the survey, with 53.54% male and 46.46% female (see Table 5). The average age of the respondents was 70.2, with the highest proportion of participants in the 66–75 age group (57.7%). Education levels varied, with 55.3% of participants having completed high school or technical school, while 38.6% had not received secondary education. Most respondents were married (88.38%) and living with family (91.92%). In terms of financial status, 72.69% of respondents reported a monthly income between 3,500 and 6,500 RMB, and 9.08% earned below 3,500 RMB, suggesting moderate economic diversity.

**Table 5:** Characteristics of older adult residents by hesitancy status.

Types of factors	Factors	Level	Total *n* (%)	No hesitancy (*n* = 190)	Vaccine hesitancy			*p*-value
					Acceptance with doubts (*n* = 310)	Refusal with doubts (*n* = 590)	Refusal (*n* = 210)	
Demographic factors	Gender							
		Female	604 (46.46)	89	143	264	108	0.42
		Male	696 (53.54)	101	167	326	102	
	Age group							
		60–65	310 (23.80)	45	81	141	43	0.5
		66–75	750 (57.70)	107	183	338	122	
		76–85	199 (15.30)	31	39	95	34	
		>85	41 (3.15)	7	7	16	11	
	Religious belief							
		Yes	99 (7.60)	20	41	24	14	<0.05*
		No	1,201 (92.40)	170	269	566	196	
	Education							
		Middle school and below	502 (38.61)	77	116	209	100	<0.05*
		High school and tech school	719 (55.30)	99	175	349	96	
		Bachelor and above	79 (6.09)	14	19	32	14	
	Marital status							
		Married	1,149 (88.39)	168	274	532	175	<0.05*
		Unmarried	28 (2.15)	3	3	10	12	
		Divorced or widowed	123 (9.46)	19	33	48	23	
	Living alone							
		Yes	105 (8.08)	14	24	40	27	<0.05*
		No	1,195 (91.92)	176	286	550	183	
	Previously worked in the medical field							
		Yes	65 (5.00)	14	16	25	10	0.39
		No	1,235 (95.00)	176	294	565	200	
	Monthly Income (CNY)							
		<3,500	118 (9.08)	23	18	49	28	<0.05*
		3,500–6,500	945 (72.69)	129	220	446	150	
		6,500–10,000	210 (16.15)	34	63	84	29	
		>10,000	27 (2.08)	4	9	11	3	
Self-report health status	History of falling							<0.05*
		Yes	79 (6.10)	13	13	31	22	
		No	1,221 (93.90)	177	297	559	188	
	Chronic disease							0.070
		Yes	800 (61.50)	120	192	348	140	
		No	500 (38.50)	70	118	242	70	
	Taken physical exam previous year							
		Yes	956 (73.54)	147	246	444	119	<0.05*
		No	344 (26.46)	43	64	146	91	
	Self-reported health status							
		Good	462 (35.50)	82	118	188	74	<0.05*
		Normal	732 (56.30)	97	167	361	107	
		Poor	106 (8.20)	11	25	41	29	
	Experience of influenza-like symptoms							
		Yes	843 (64.80)	94	175	414	160	<0.05*
		No	457 (35.20)	96	135	176	50	
Psychological factors	GDS-15 score							
		≥5	275 (21.1)	21	49	138	67	<0.05*
		<5	1,025 (78.9)	169	261	452	143	
	Needle phobia							
		Low	510 (39.20)	98	138	201	73	<0.05*
		Moderate	484 (37.20)	60	113	236	75	
		High	306 (23.60)	32	59	153	62	
	Fear of vaccination side-effects							
		Low	285 (21.90)	67	88	103	27	<0.05*
		Moderate	441 (33.90)	55	111	210	65	
		High	574 (44.20)	68	111	277	118	
Self-report vaccination experiences	Covid vaccine history							
		Yes	1,075 (82.69)	181	295	488	111	<0.05*
		No	225 (17.30)	9	15	102	99	
	Influenza vaccine history							
		Yes	231 (17.77)	122	60	39	10	<0.05*
		No	1,069 (82.23)	68	250	551	200	
	PPSV23 vaccine history							
		Yes	147 (11.30)	51	44	45	7	<0.05*
		No	1,153 (88.69)	139	266	545	203	
	Vaccination side-effect experience							
		Yes	54 (4.15)	2	7	30	15	<0.05*
		No	1,246 (95.85)	188	303	560	195	

PPSV23, 23-valent pneumococcal polysaccharide vaccine. *p-value <0.05 is considered statistically significant.

Health-related characteristics also varied within the sample. 73.54% of the older adult had undergone physical examinations in the past year, and 61.4% reported having chronic diseases. 21.1% of the older adult population scored five or above on the GDS-15, suggesting a potential predisposition to depression. Most respondents self-rated their health status as normal (56.3%) and relatively good (35.5%), while 8.2% perceived their health as poor. 64.8% had experienced influenza-like symptoms in the past year, and VH was significantly higher in this group (*p* < 0.05). A considerable proportion of participants reported needle phobia (60.8%) and concerns about vaccine side effects (78.1%), both of which were strongly associated with hesitancy (*p* < 0.05). Vaccination history showed high coverage of COVID-19 vaccines (82.69%), exceeding the WHO target of ≥70% coverage for total populations by mid-2022 (42). While only 17.77% had received an influenza vaccine, and 11.3% had received a 23-valent pneumococcal polysaccharide vaccine (PPSV23). Although most participants reported no direct side effects from vaccination, 15% indicated hearing negative information about vaccines from neighbors, which contributed to greater hesitancy (*p* < 0.05). Regarding engagement with healthcare providers, 73% of the older adult had registered with a family doctor, yet over 50% reported infrequent health communication with their physicians. Additionally, over half of the respondents did not actively enquire about influenza vaccination with their family doctors, indicating potential gaps in vaccine promotion (*p* < 0.05).

### 3.3 Determinants of influenza VH

#### 3.3.1 Confidence factors

73.3% of respondents trusted health workers, with higher trust levels observed among those without hesitancy (*n* = 176) compared to refusers (*n* = 125). Similarly, trust in the efficacy of influenza vaccines was reported by 54.08% of participants, with hesitancy refusers showing the lowest trust levels (*n* = 52). All the factors of convenience present significant difference among different hesitancy status groups (*p* < 0.05).

Table 6 presents the multinomial logistic regression results identifying factors associated with VH. Distrust in the efficacy of influenza vaccines emerged as the strongest predictor of hesitancy. Compared to individuals who trusted the influenza vaccine, those who distrusted its efficacy had 2.28 times greater odds of vaccine refusal (95% CI: 1.12–6.38) and 3.42 times greater odds of accepting it with doubts (95% CI: 1.34–5.59). Those who distrust the safety of influenza vaccines tended to refuse influenza vaccines (aOR = 3.16, 95% CI:1.43–7.41).

**Table 6:** Multinomial logistic regression to identify factors associated with influenza vaccine hesitancy.

Types of determinants	Determinants	Level	Refusal and refusal with doubts aOR (95%CI)	Acceptance with doubts aOR (95%CI)
Confidence	Trust in CHWs	(Rf: Trust)		
		Distrust	4.29 (0.95, 19.3)	2.92 (0.05, 15.76)
	Trust in efficacy of influenza vaccines	(Rf: Trust)		
		Distrust	2.28 (1.12, 6.38)	3.42 (1.34, 5.59)
	Trust in safety of influenza vaccines	(Rf: Trust)		
		Distrust	3.16 (1.43, 7.41)	1.34 (0.22,8.23)
Complacency	Perceived severity toward influenza	(Rf: High)		
		Low	2.36 (0.87, 6.4)	2.75 (1.12, 6.75)
	Perceived severity toward co-infection	(Rf: High)		
		Low	0.33 (0.09, 1.13)	0.27 (0.08, 1.84)
	Perceived severity of spreading influenza to family	(Rf: High)		
		Low	5.59 (1.46, 21.37)	1.85 (0.49, 7)
	Susceptibility to influenza	(Rf: High)		
		Low	1.65 (0.45, 6.1)	1.33 (0.33, 5.39)
	Susceptibility to co-infection	(Rf: High)		
		Low	1.3 (0.41, 4.1)	1.33 (0.41,4.29)
	Perceived necessity due to influenza-specific medication	(Rf: High)		
		Low	3.76 (2.55, 5.53)	1.76 (0.53, 5.84)
	Perceived necessity due to trust in immune system	(Rf: High)		
		Low	0.25 (0.04, 1.47)	1.94 (0.6, 6.3)
	Perceived necessity of influenza vaccination among older adult people	(Rf: High)		
		Low	0.87 (0.18,4.32)	2.55 (1.01, 6.4)
	Perceived necessity due to NPIs	(Rf: High)		
		Low	3.37 (1.21, 9.38)	0.7 (0.27, 1.81)
Convenience	Vaccination independently	(Rf: Agree)		
		Moderate	1.39 (0.52, 3.67)	1.36 (0.54, 3.44)
		Disagree	4.96 (1.18, 20.77)	0.89 (0.2, 3.87)
	Family support	(Rf: Sufficient)		
		Moderate	3.72 (1.56, 8.87)	3.38 (1.57, 7.28)
		Insufficient	3.63 (1.12, 11.82)	3.42 (1.97, 12.09)
	Health communication with CHWs	(Rf: Frequent)		
		Not often	0.41 (0.17, 1.04)	1.03 (0.42, 2.38)
		Never	0.26 (0.09, 0.74)	0.37 (0.13, 1.02)
	Vaccine-related health communication with CHWs	(Rf: Frequent)		
		Not often	0.83 (0.33, 2.05)	1.6 (0.6, 4.25)
		Never	0.19 (0.06, 0.66)	1.88 (0.38, 9.26)
	Have an assigned family physician	(Rf: No)		
		Yes	1.31 (0.52, 3.27)	1.54 (0.69, 3.45)
			
			
Vaccine Literacy	Vaccine knowledge	(Rf: High)		
		Moderate	1.52 (0.68, 3.41)	2.09 (1.08, 4.06)
		Low	1.51 (0.63, 3.63)	1.85 (1.73, 4.41)
	Functional vaccine literacy		0.64 (0.4, 1.02)	0.57 (0.38,1.85)
	Interactive vaccine literacy		1.26 (0.6, 2.64)	1.04 (0.51, 2.11)
	Critical vaccine literacy		0.36 (0.29, 0.45)	0.6 (0.32, 1.15)
Psychological factors	Needle phobia	(Rf: Low)		
		Moderate	0.92 (0.4, 2.11)	1.09 (0.54, 2.22)
		High	3.93 (1.51, 10.25)	0.91 (0.36, 2.29)
	Fear of the side effects after vaccination	(Rf: Low)		
		Moderate	0.9 (0.34, 2.42)	0.61 (0.27, 1.37)
		High	0.74 (0.3, 1.82)	0.3 (0.13, 1.69)
	GDS-15 score	(Rf: <5)		
		≥5	2.72 (1.14, 6.53)	1.93 (0.82, 4.56)

aOR, adjusted odds ratio; CI, confidence interval; CHWs, community health workers; PPSV23, 23-valent pneumococcal polysaccharide vaccine; NPIs, non-pharmaceutical interventions, adjustment set: sociodemographic variables, self-report health status and self-report vaccination experiences.

#### 3.3.2 Complacency and perceived risk

**Figure 2:**
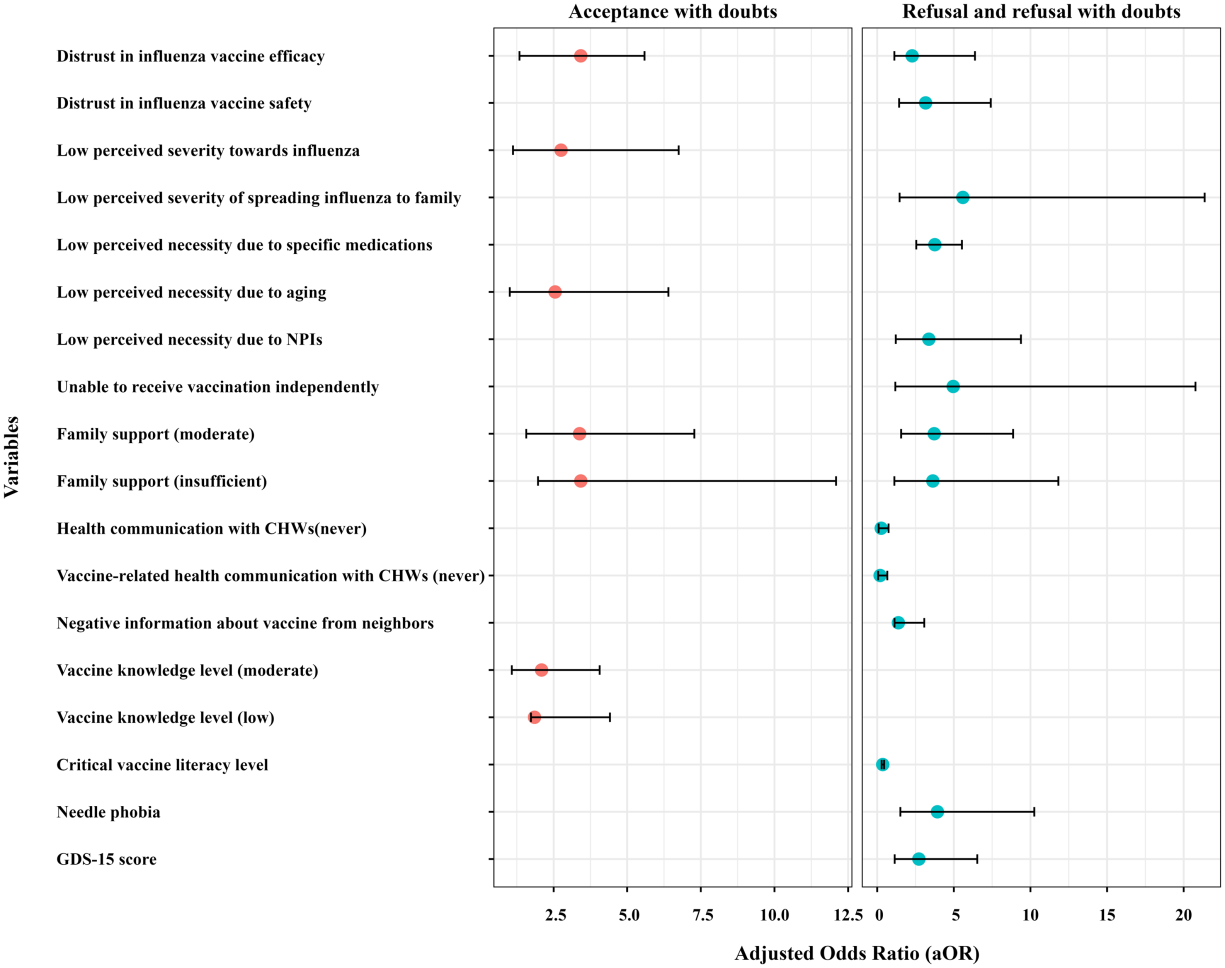
Forest plot comparing the adjusted odds ratio (aOR) values of influenza vaccine hesitancy. aOR, adjusted odds ratio; CHWs, community health workers; NPIs, non-pharmaceutical interventions; GDS, Geriatric Depression Scale.

Perceived risk and necessity of vaccination influenced VH. 44.77% of participants perceived influenza as highly severe, with higher proportions in the non-hesitant group (*n* = 120) compared to refusers (*n* = 81). Additionally, the perceived necessity of vaccination due to trust in the immune system or the availability of influenza-specific medicines showed significant variation across hesitancy groups. Table 6 demonstrates that vaccine acceptors with doubts exhibited both significantly lower risk perception regarding influenza severity (aOR = 2.75; 95% CI:1.12–6.75) and higher endorsement of age-related vaccination misconceptions (aOR = 2.55; 95% CI:1.01–6.40) compared to their non-hesitant older adult. Those who believed that influenza infection posed a low risk to family members were significantly more likely to be vaccine refusers (aOR = 5.59, 95% CI:1.46–21.37). Similarly, the perceived low necessity of vaccination due to the availability of influenza-specific medications increased refusal rates (aOR = 3.76, 95% CI:2.55–5.53). Those who believed that NPIs such as masks and social distancing were sufficient for protection had 3.37 times greater odds of refusing vaccination (95% CI: 1.21–9.38). However, these factors did not influence acceptors with hesitancy (see Figure 2).

#### 3.3.3 Convenience and healthcare access

Barriers to vaccine access and support networks also influenced hesitancy. 56.31% of participants reported the ability to vaccinate independently, with higher proportions in the non-hesitant group (*n* = 160) than in refusers (*n* = 82). Family support also played a critical role, with 34.15% reporting no support, predominantly among non-hesitant individuals (*n* = 143). Multinomial regression in Table 6 confirmed that lack of family support was a major factor, with participants who reported moderate family support having 3.72 times greater odds of vaccine refusal (95% CI: 1.56–8.87), while those with no family support had 3.63 times higher odds of acceptance with hesitancy (95% CI: 1.12–11.82). Insufficient family support might also cause older adults to be vaccine acceptors with doubts (aOR = 3.38, 95% CI: 1.57–7.28; aOR = 3.42, 95% CI: 1.97–12.09). Vaccine refusers demonstrated substantially reduced capacity for autonomous vaccination-site access compared to vaccine acceptors (aOR = 4.96; 95% CI:1.18–20.77), indicating significant functional mobility barriers in achieving vaccination compliance.

Compared to those who frequently interacted with healthcare providers, the older adult who never engaged in health communication had significantly lower odds of refusal (aOR = 0.26, 95% CI: 0.09–0.74) and accepting vaccines with doubts (aOR = 0.37, 95% CI: 0.13–1.02). Similarly, individuals who never had vaccine-related health communication with CHWs were less likely to refuse influenza vaccines (aOR = 0.19, 95% CI: 0.06–0.66). Older adults who had heard of vaccine-related adverse events from family members or neighbors were more likely to be vaccine refusers (aOR = 1.38; 95% CI: 1.13, 3.07).

### 3.4 Association between VL and VH

**Figure 3:**
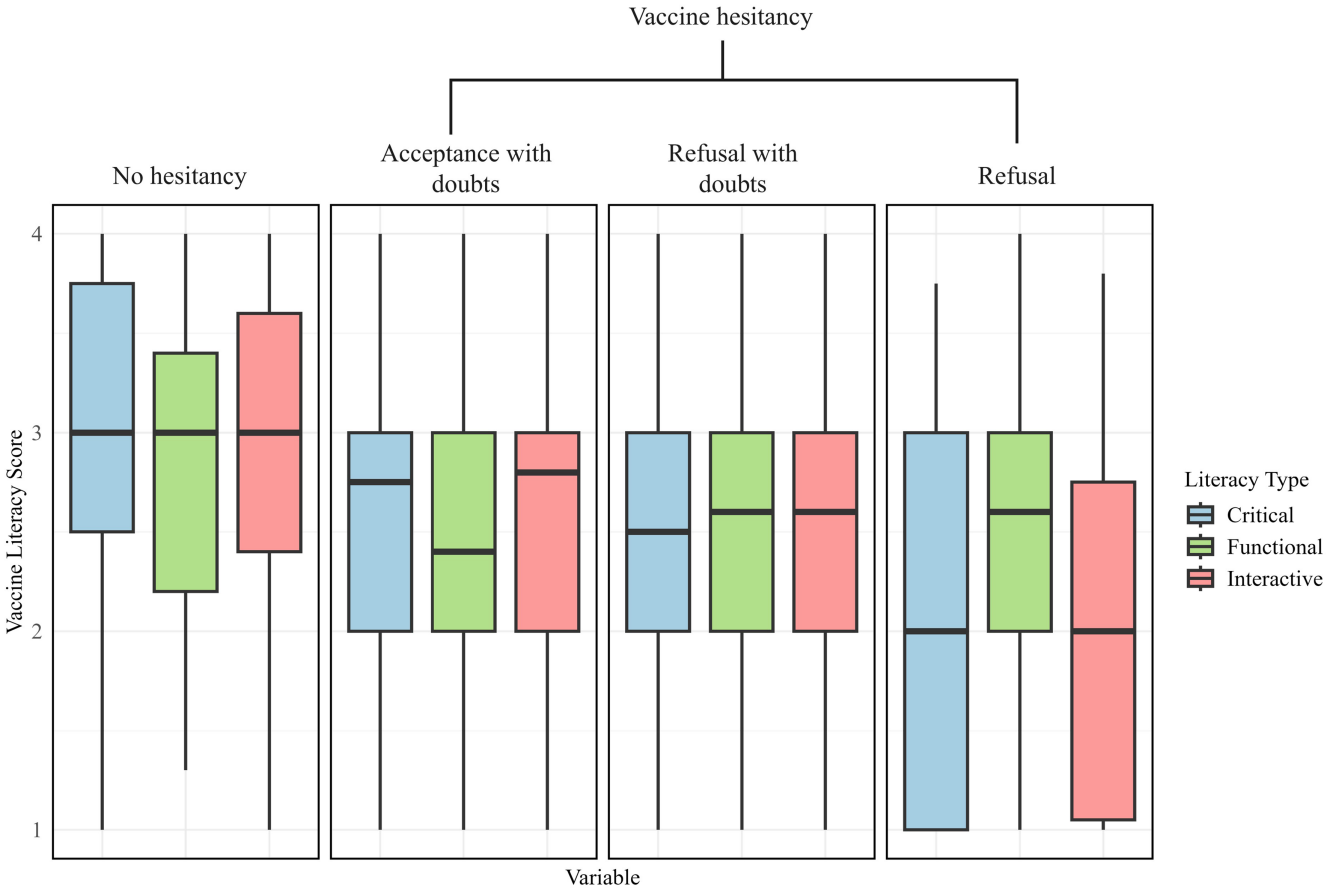
The boxplot of the older adult’s vaccine literacy scores across the three literacy types: critical, functional and interactive.

Only 21.62% of respondents demonstrated high vaccine knowledge, with higher proportions in the non-hesitant group (*n* = 74) compared to refusers (*n* = 35). Conversely, 39.3% had low vaccine knowledge, with the highest proportion among hesitancy refusers (*n* = 110). Figure 3 displays the distribution of VL scores across different hesitancy status. Participants with no hesitancy exhibited the highest scores across all three domains of VL. Functional literacy remained relatively stable, but interactive and critical VL levels were significantly lower in refusers, indicating difficulty in analyzing and interpreting vaccine-related information. Regression analysis (see Table 6) confirmed that lower vaccine knowledge significantly increased hesitancy. Individuals with moderate vaccine knowledge were 2.09 times more likely to hesitate (95% CI: 1.08–4.06), while those with low knowledge had 1.85 times greater odds (95% CI: 1.73–4.41). Additionally, the older adult who had higher critical vaccination literacy were more likely to refuse influenza vaccines (aOR = 0.36, 95% CI: 0.29–0.45).

### 3.5 Psychological factors

Older adults who had needle phobia (aOR = 3.93; 95% CI: 1.51, 10.25) were more likely to be vaccine refusal. Also, the effect of potential depression symptoms was more pronounced in the vaccine refusal older adult population (aOR = 2.72; 95% CI: 1.14, 6.53).

### 3.6 SEM with 3Cs factors and VL

**Figure 4:**
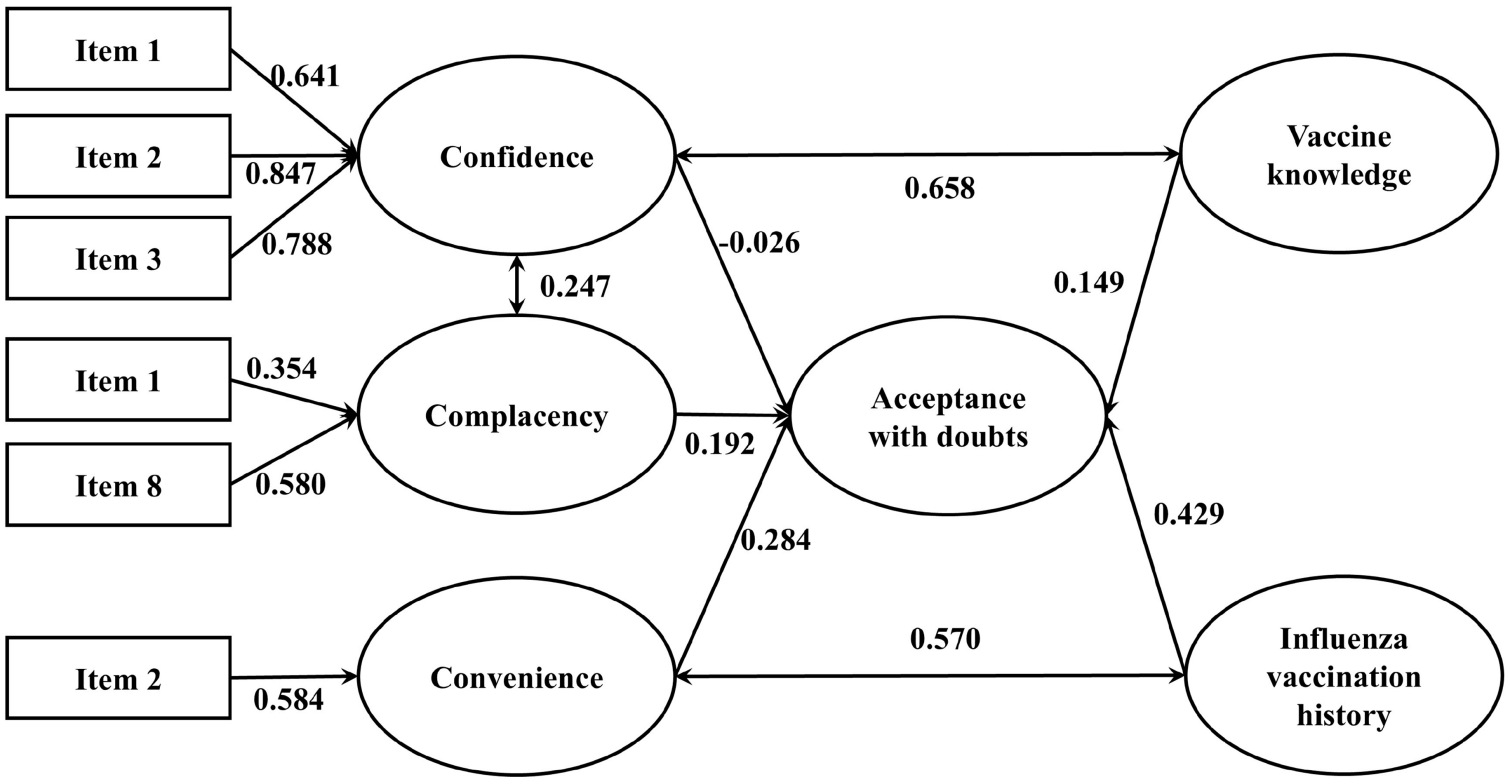
The integrated SEM with 3Cs effects and vaccine literacy on influenza VH (Acceptance with doubts). *p*-value <0.05 is considered statistically significant. Only results that have significant effects are presented. *N* = 1,300.

**Figure 5:**
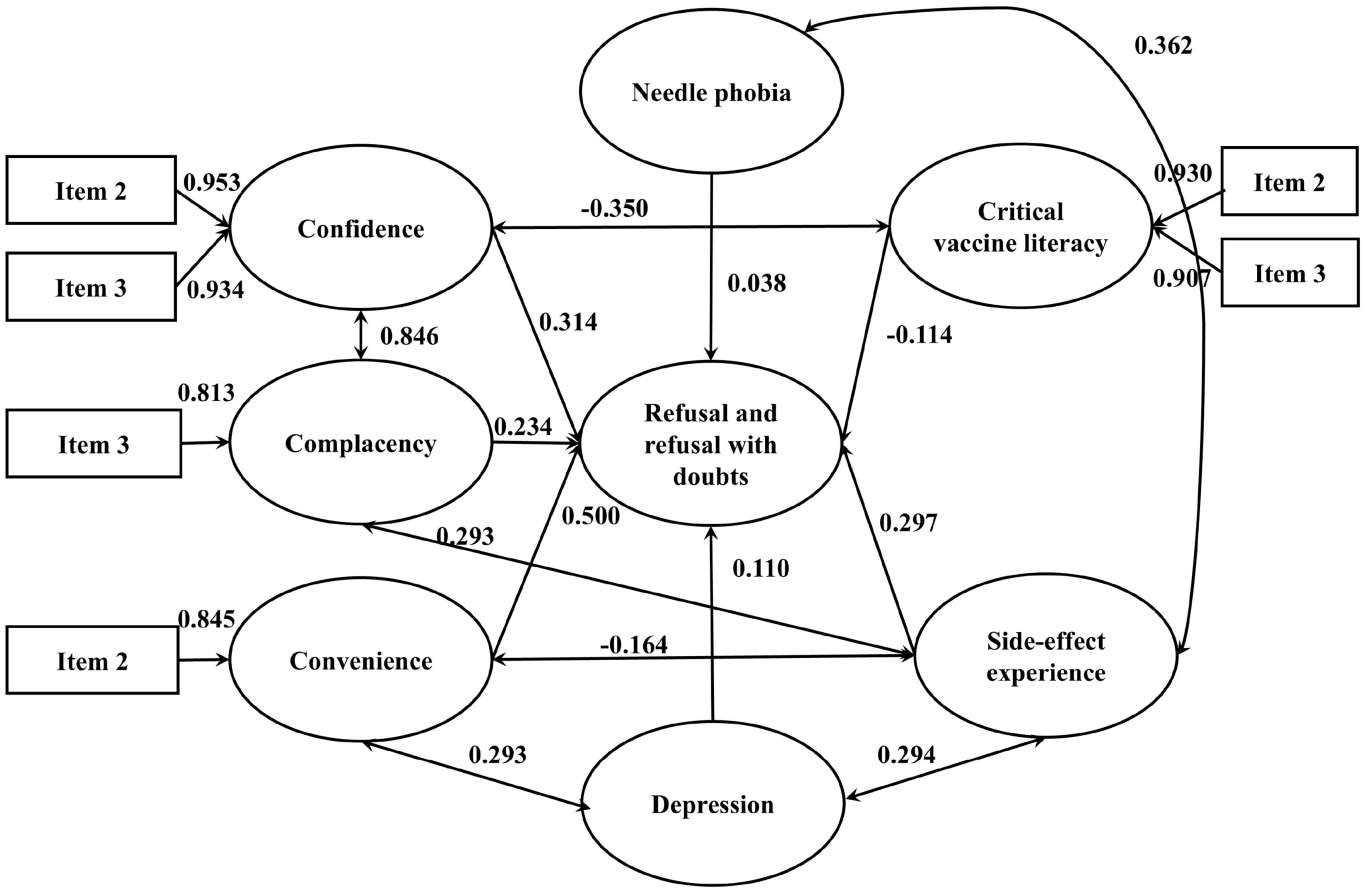
The integrated SEM with 3Cs effects and vaccine literacy on influenza VH (Refusal and refusal with doubts). *p*-value <0.05 is considered statistically significant. Only results that have significant effects are presented. *N* = 1,300.

Table 5 presents the goodness-of-fit indices for the integrated model of influenza vaccination hesitancy. All indices fell within acceptable ranges, indicating adequate model fit (43). Each pathway was statistically significant using standardized estimated coefficient (*β*) based on the best final integrated model (see Figures 4, 5).

Among vaccine acceptors with hesitancy, confidence (*β* = −0.026), convenience (*β* = 0.284) and complacency (*β* = 0.192) all significantly and directly affected VH. Vaccination history and vaccine knowledge also contribute positively to vaccine hesitancy, with standardized coefficients of 0.429 and 0.149, respectively. There were significant covariances between confidence and complacency (*β* = 0.247), vaccine knowledge and confidence (*β* = 0.658), influenza vaccination history and convenience (*β* = 0.570). Among vaccine refusers, confidence (*β* = 0.314), convenience (*β* = 0.5) and complacency (*β* = 0.234) all significantly and directly affected vaccine refusal. Depression, side-effect experience and needle phobia also contribute positively to vaccine hesitancy, with standardized coefficients of 0.11, 0.297 and 0.038, respectively. Critical vaccine literacy negatively affected vaccine refusal (*β* = −0.114) and confidence (*β* = −0.350). There were significant covariances between confidence and complacency (*β* = 0.846), side-effect experience and complacency (*β* = 0.293), side-effect experience and depression (*β* = 0.294), convenience and depression (*β* = 0.293), side-effect experience and needle phobia (*β* = 0.362).

### 3.7 VH toward influenza and COVID-19

**Figure 6:**
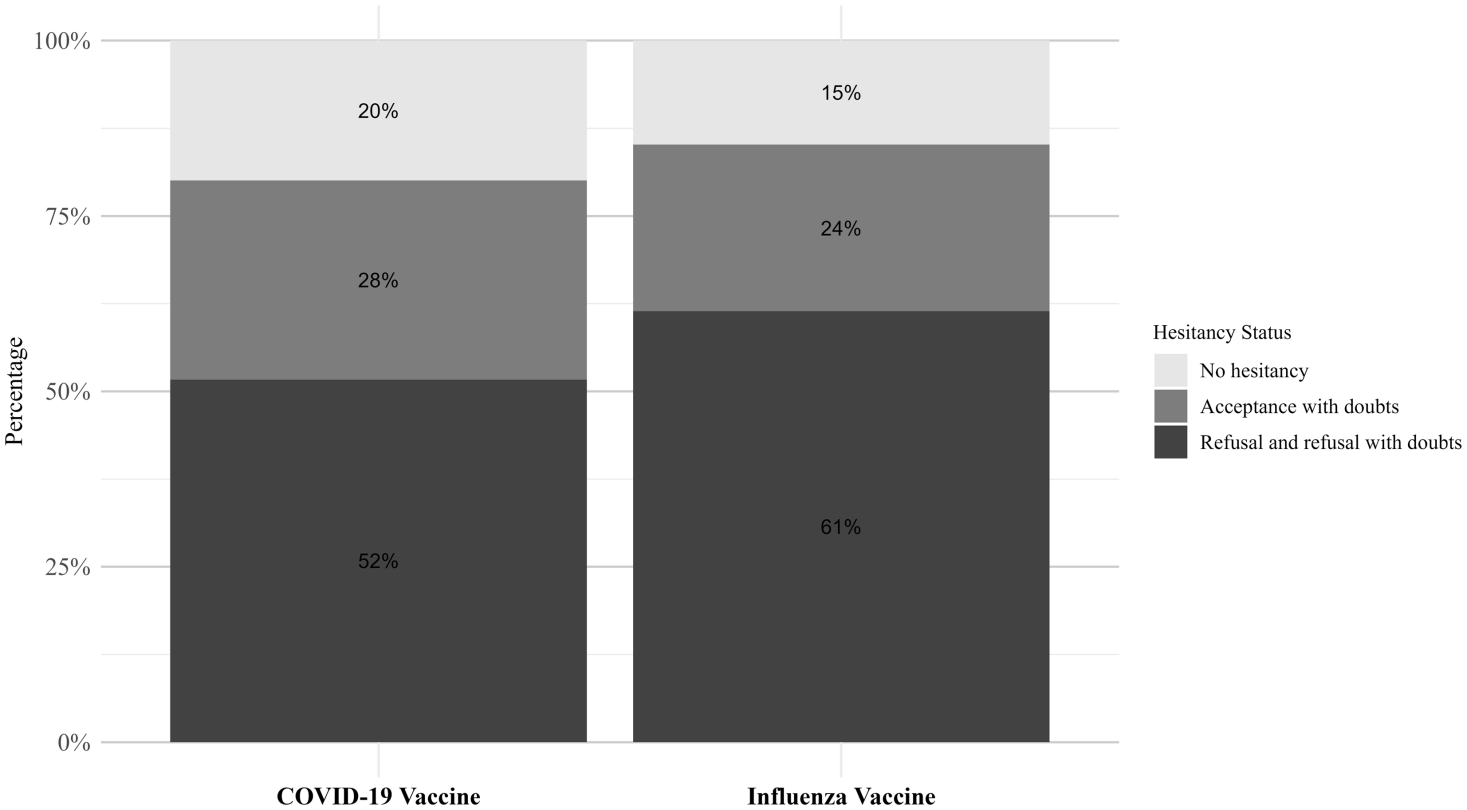
The distribution of hesitancy status among the older adult regarding the COVID-19 and influenza vaccines.

Figure 6 illustrates the distribution of VH status for influenza and COVID-19 vaccines. Among all participants, the proportion of non-hesitators (those who fully accepted the vaccine) was lower for influenza (14.8%) compared to COVID-19 (19.92%), suggesting greater reluctance toward influenza vaccination. The largest subgroup among both vaccines was refusers with doubts, accounting for 45.2% for influenza and 37.0% for COVID-19. Absolute refusers were also slightly more prevalent for influenza (16.2%) than for COVID-19 (14.68%), reinforcing the stronger hesitancy toward influenza vaccines.

## 4 Discussion

This study offers a detailed examination of influenza VH among older adults in Shanghai, China, utilizing the 3Cs model and VL framework to uncover the multifaceted determinants shaping vaccine decision-making in this population. The findings reveal a stark contrast between hesitancy toward influenza and COVID-19 vaccines, suggesting that external contextual factors, alongside psychological and structural influences, play a critical role. By applying the 3Cs model and incorporating VL as an additional determinant, this study offers novel insights into the multi-dimensional factors influencing vaccine decision-making in the older adult population.

### 4.1 Key findings

The 3Cs framework illuminated the central role of confidence, complacency, and convenience in driving influenza VH. A lack of trust in vaccine effectiveness and safety emerged as a key barrier, reflecting broader concerns about healthcare systems and information credibility. Complacency was evident in the underestimation of influenza’s risks, particularly its potential to affect family members, which was further compounded by reliance on alternative preventive measures. Convenience issues, such as limited family support and access to vaccination services, also hindered uptake.

Notably, critical VL emerged as a dual-force factor negatively influencing vaccine refusal and confidence, underscoring the necessity for customized literacy interventions that target specific drivers of vaccine hesitancy. Additionally, the study identified a significant influence from COVID-19 VH spillover, likely stemming from the high acceptance of COVID-19 vaccines compared to influenza vaccines. This spillover appears to shape perceptions of disease severity and necessity, aligning with the complacency dimension, and underscores the interconnected nature of vaccine attitudes in the post-pandemic era (44).

### 4.2 Confidence in influenza vaccination

Confidence in vaccine efficacy emerged as the strongest predictor of hesitancy, reinforcing previous research that trust in vaccines and healthcare providers is a crucial determinant of vaccine acceptance (45, 46). Participants who distrusted the effectiveness of influenza vaccines were over 25 times more likely to refuse vaccination, a pattern not observed for COVID-19 vaccines. Unlike the widespread trust fostered by COVID-19 vaccination campaigns, skepticism toward influenza vaccines persists, possibly due to lingering misinformation or historical perceptions of vaccine efficacy (47–49).

Additionally, concerns about vaccine safety played a role, though its impact was less pronounced than efficacy-related concerns. This aligns with prior studies indicating that, among older adults, the perceived effectiveness of vaccination influences decision-making more than safety concerns (50). Addressing these concerns requires targeted communication strategies that reinforce trust while acknowledging safety considerations, potentially leveraging community health workers to bridge the confidence gap.

### 4.3 Complacency and the perceived risk of influenza

Complacency emerged as a significant contributor to VH, driven by a tendency to downplay the severity of influenza and its complications, especially within family contexts. This perception was exacerbated by the availability of influenza-specific medications and the belief that NPIs, such as wearing masks or social distancing, provided sufficient protection. Previous studies have highlighted similar patterns, where low perceived risk reduces the motivation for vaccination (51). The post-COVID-19 context further complicates this, as the heightened focus on COVID-19 may have diminished attention to influenza risks, including the dangers of co-infection (5). Given the ongoing circulation of both viruses, public health messaging should emphasize the added risks of co-infection and highlight how influenza vaccination complements COVID-19 vaccines in reducing disease burden. Moreover, older adults who accept vaccines with doubts were more likely to mistakenly believe vaccines become unnecessary with age (e.g., “I’ve lived long enough”; aOR = 2.55, 95% CI:1.01–6.40). Public health efforts should therefore emphasize the cumulative risks of influenza and the complementary role of vaccination, particularly for older adults who may misjudge the benefits due to age-related assumptions.

### 4.4 VL-3Cs interactions

Despite VL’s theoretical importance, its role in directly influencing influenza VH was limited, prompting a deeper exploration of its interactions with the 3Cs dimensions. Functional VL, which involves basic comprehension of vaccine information, did not independently reduce hesitancy, possibly because basic knowledge alone does not address deeper attitudinal barriers like distrust (28). However, functional VL likely interacts with confidence, as improved understanding of vaccine benefits could enhance trust in efficacy, though this effect may have been overshadowed by the pandemic context. Interactive VL, which involves seeking and discussing vaccine information, showed a nuanced effect, appearing more relevant among acceptors with doubts, potentially by facilitating engagement with CHWs and reducing uncertainty (52). Unexpectedly, higher critical VL, which reflects the ability to critically evaluate vaccine information, was associated with greater hesitancy, possibly due to increased skepticism or susceptibility to misinformation among those who critically appraise health messages (53, 54). This suggests a complex interaction with complacency, as critical evaluators may be more likely to endorse alternative protective measures like NPIs. These VL-3Cs interactions indicate that VL’s impact is not uniform but varies depending on the attitudinal and contextual factors captured by the 3Cs model, highlighting the need for tailored literacy interventions that address specific hesitancy drivers.

### 4.5 Convenience and healthcare system barriers

Structural barriers significantly influenced VH, with inadequate family support and limited access to vaccination services posing substantial obstacles. The role of social networks in decision-making aligns with previous research, highlighting the need for family involvement in health interventions (55). Interestingly, the lack of engagement with community health workers (CHWs) appeared to have a complex effect, potentially indicating that refusers sought information from alternative sources (e.g., social media platforms or interpersonal networks with peers/relatives). This is particularly concerning, given that over 50% of participants had infrequent or no discussions about influenza vaccination with their family doctors. This gap underscores the compounding role of provider engagement: prior research has demonstrated that consistent, clinician-led counseling remains a pivotal driver of vaccine confidence (56). To disrupt this cycle, healthcare systems must mandate provider-initiated vaccine dialogues during clinical encounters, particularly for high-risk groups with fragmented access to credible health information. Embedding such discussions into standard care protocols could transform passive patient-provider interactions into trust-building opportunities, ensuring that vaccine hesitancy is preemptively addressed rather than passively observed.

### 4.6 The spillover effects: the link between COVID-19 and influenza VH

The negative spillover from COVID-19 VH significantly shaped influenza VH, as the higher trust in COVID-19 vaccines did not translate into confidence in influenza vaccines (57). This spillover likely amplified complacency toward influenza, as older adults prioritized COVID-19 risks, underestimating influenza’s threat. The distinct hesitancy patterns-influenza vaccines facing more outright refusal compared to COVID-19 vaccines attracting more hesitant acceptors-suggest that lessons from COVID-19 vaccine campaigns, such as government transparency and media outreach, could be adapted to improve influenza vaccine uptake.

### 4.7 Policy implications and recommendation

1. Targeting Vaccine Confidence: Campaigns should prioritize building trust in vaccine efficacy through transparent, evidence-based communication.
2. Addressing Complacency: Messaging should emphasize influenza’s severity, particularly co-infection risks, and the limitations of alternative measures.
3. Enhancing Healthcare Engagement: Train CHWs to initiate vaccine discussions during routine visits, involving family members to bolster support.
4. Strengthening VL: Develop literacy programs that not only improve knowledge but also enhance critical appraisal skills to counter misinformation.
5. Tailored Interventions: Differentiate strategies for refusers and acceptors with doubts, addressing their unique concerns.
6. Psychological support: Integrate vaccine psychology screening into primary care (depression GDS-15 ≥ 5, prior side-effect trauma, or needle phobia) and deliver first-line cognitive behavioral therapy to the older adult.
7. Mitigating Spillover Effects: Leverage COVID-19 vaccine trust through integrated vaccine promotion strategies.
8. Assessing Financial Barriers: Explore the role of financial incentives in improving uptake, despite cost being a minor factor in this study.

### 4.8 Strengths and limitations

This study has several strengths. It is one of the first studies in China to examine influenza VH using the 3Cs model alongside VL factors. The large, community-based sample enhances the generalizability of the findings. Additionally, the inclusion of COVID-19 VH as a comparison provides important insights into the broader vaccine confidence landscape. However, some limitations should be acknowledged. This study was conducted exclusively in Shanghai, a metropolitan area with higher socioeconomic status and advanced healthcare infrastructure. The findings may not fully represent populations in rural regions where distinct challenges exist, including differential access to healthcare services and variations in vaccine literacy levels. Caution should therefore be exercised when generalizing these results to non-urban settings. Future multi-regional studies are needed to provide a more comprehensive understanding of the older adult’s hesitancy toward influenza vaccines across China’s diverse populations. Additionally, the cross-sectional nature of the study prevents causal inferences about VH determinants. Future longitudinal studies are needed to assess changes in vaccine attitudes over time, particularly in the evolving post-pandemic landscape.

## 5 Conclusion

Influenza VH among older adults in Shanghai remains a significant challenge, driven by low confidence, complacency, and convenience barriers. While VL’s direct impact was limited, its interactions with the 3Cs suggest a nuanced role in shaping hesitancy, moderated by the COVID-19 VH spillover. Integrated public health strategies that address these interconnected factors, counter misinformation, and enhance healthcare engagement are essential to improve vaccine uptake in this high-risk population.

## Data Availability

The datasets presented in this article are not readily available because the authors do not have permission to share data. Requests to access the datasets should be directed to alvajerry@163.com.

## Footnotes

^1^Wenjuanxing, https://www.wjx.cn.
